# Identification of enhanced hydrogen and ethanol *Escherichia coli* producer strains in a glycerol-based medium by screening in single-knock out mutant collections

**DOI:** 10.1186/s12934-015-0285-6

**Published:** 2015-06-28

**Authors:** Antonio Valle, Gema Cabrera, Domingo Cantero, Jorge Bolivar

**Affiliations:** Department of Biomedicine, Biotechnology and Public Health-Biochemistry and Molecular Biology, Campus de Excelencia Internacional Agroalimentario (ceiA3), Institute of Biomolecules, University of Cádiz, Avda República Saharui s/n, 11510 Puerto Real, Cádiz Spain; Department of Chemical Engineering and Food Technology, Campus de Excelencia Internacional Agroalimentario (ceiA3), University of Cádiz, Avda República Saharui s/n, 11510 Puerto Real, Cádiz Spain

**Keywords:** *Escherichia coli*, Ethanol, Glycerol, High throughput, Hydrogen

## Abstract

**Background:**

Earth’s climate is warming as a result of anthropogenic emissions of greenhouse gases from fossil fuel combustion. Bioenergy, which includes biodiesel, biohydrogen and bioethanol, has emerged as a sustainable alternative fuel source. For this reason, in recent years biodiesel production has become widespread but this industry currently generates a huge amount of glycerol as a by-product, which has become an environmental problem in its own right. A feasible possibility to solve this problem is the use of waste glycerol as a carbon source for microbial transformation into biofuels such as hydrogen and ethanol. For instance, *Escherichia coli* is a microorganism that can synthesize these compounds under anaerobic conditions.

**Results:**

In this work an experimental procedure was established for screening *E. coli* single mutants to identify strains with enhanced ethanol and/or H_2_ productions compared to the wild type strain. In an initial screening of 150 single mutants, 12 novel strains (*gnd*, *tdcE*, *rpiAnanE*, *tdcB*, *deoB*, *sucB*, *cpsG*, *frmA*, *glgC*, *fumA* and *gadB*) were found to provide enhanced yields for at least one of the target products. The mutations, that improve most significantly the parameters evaluated (*gnd* and *tdcE* genes), were combined with other mutations in three engineered *E. coli* mutant strains in order to further redirect carbon flux towards the desired products.

**Conclusions:**

This methodology can be a useful tool to disclose the metabolic pathways that are more susceptible to manipulation in order to obtain higher molar yields of hydrogen and ethanol using glycerol as main carbon source in multiple *E. coli* mutants.

**Electronic supplementary material:**

The online version of this article (doi:10.1186/s12934-015-0285-6) contains supplementary material, which is available to authorized users.

## Background

Earth’s climate is warming as a result of anthropogenic emissions of greenhouse gases, particularly carbon dioxide (CO_2_) from fossil fuel combustion. Significant opportunities to mitigate anthropogenic emissions of these gases exist, although some will be easier to exploit than others [1]. Recently, the production and use of biofuels like biodiesel and bioethanol have become widespread because they are more sustainable, secure, renewable, and environmentally safe than the fossil fuels [2, 3]. Hydrogen (H_2_) is also universally recognized as an environmental friendly and safe renewable resource [4]. Therefore, the production of H_2_ and ethanol from biomass will probably play an important role in bioenergy generation [5]. However, although biodiesel production has emerged as a possible alternative to fossil fuels, this industry generates glycerol as a by-product in such large quantities that its market value has dropped and it has become an environmental problem in its own right. For these reasons, several practical processes for the conversion of glycerol into high-value products have been proposed [6]. In this regard, glycerol represents a cheap carbon source that has been used in several biotransformation processes for the production of added-value products [2, 7–16] including the generation of hydrogen and ethanol [17, 18].

*Escherichia coli*—one of the most commonly host organism used for metabolic engineering and biotechnological applications [19–21]—is a suitable species for glycerol utilization either under aerobic or anaerobic conditions [14, 22]. The fermentation of glycerol starts with its conversion to DHAP, which is mediated by a two-branch pathway: the oxidative branch by glycerol dehydrogenase and dihydroxyacetone kinase enzymes; and the reductive branch by glycerol kinase and glycerol 3-phosphate dehydrogenase enzymes. Then DHAP can be then metabolized in the glycolysis pathway to pyruvate. In this process 2 NAD^+^ are reduced, one of them in the assimilation of glycerol and another one in the synthesis of 1,3 bisphosphoglycerate.

Ethanol and H_2_ are originated in *E. coli* after the anaerobic mixed-acid fermentation in which pyruvate, the final product of glycolysis, is fermented to formate, the substrate for H_2_ synthesis, and acetyl-CoA, which after two reductive steps is transformed to ethanol in which two NADH are re-oxidized balancing in this way the NADH/NAD^+^ ratio during the assimilation of glycerol [14, 22].

Several factors regulate the production of hydrogen and ethanol. Among them: pH, metabolites transport from or into the cell [23, 24], and the carbon source. In this sense, it has been described that hydrogen production by the hydrogenase enzymes (Hyd-1, 2, 3 and 4) in *E. coli* is influenced by pH and for instance optimal pH of activity for the Hyd-3 is around pH 6.5 [2, 25, 26]. On the other hand, glycerol metabolization is enhanced at neutral pH and is highly active at alkaline pH [22]. Several relevant studies have also revealed how the hydrogen and ethanol yields can be improved by genetic engineering in *E. coli* [22, 27–30]. For instance it has been recently described that the deletion of transporters of precursors, such as the formic transporter FocA and FocB, can improve the production of ethanol and/or H_2_ [23, 24, 31]. Other strategies have consisted on the blockage of one or several pathways involved in the synthesis of competitive products [21–24, 28, 32–34] and/or the overexpression of enzymes or transcription factors. For instance, the overexpression of genes involved in the uptake and conversion of glycerol (GldA) and/or expression of hydrogenase transcriptional factors (FhlA) can help to redirect the carbon flux towards the production of these target products [22, 24, 28, 32, 33]. Both strategies have been successfully combined and, for instance, Tran et al. [23] have described recently a multiple mutant *E. coli* strain that increases the molar yield for hydrogen and ethanol production.

Metabolic reactions operate as a network rather than linear pathways. When a microorganism is used for the bioproduction of compounds such as H_2_ and ethanol, the wild type organism often renders suboptimal and unsatisfactory yields. For this reason, metabolic engineering is an emerging field whose objective is to alter the metabolic network through genetic modification in order to improve the production of biofuels such as ethanol, butanol, propanol, biodiesel and hydrogen [35]. When an experimental design in metabolic engineering is aimed towards the production of a specific product, the resulting phenotypes are often suboptimal and unsatisfactory due to the distant effects of genetic modifications or unknown regulatory interactions [36]. In contrast, genetic high-throughput screenings can reveal unexpected genetic backgrounds that are suitable for the production of a particular desired compound. These techniques also have the potential to disclose interactions between different metabolic pathways.

In order to understand the role of the protein-encoding genes in the metabolism, it is useful to study the loss of function by analysing gene knockout phenotypes. Due to the lack of available information in databases concerning growth and multi-omics data for *E. coli* grown under anaerobic conditions in a glycerol based-medium, in the work described here a robust and reproducible experimental design has been established for screening of *E. coli* single mutant strains that allowed the characterization of H_2_ and ethanol productions together with the glycerol consumption. In this design, the use of mini-reactors under anaerobic standardized conditions coupled to automated gas chromatography (GC) and high performance liquid chromatography (HPLC) allowed the easy and reliable measurement of hydrogen, ethanol and glycerol.

In this work an initial screening of 150 single knockout strains from the Keio and Yale Collections, was carried out and 12 novel mutants with enhanced ethanol and/or H_2_ production and/or glycerol consumption respect to the *E. coli* wild type strain were found. Moreover, based on these results, several multiple mutant strains have also been engineered in order to improve the target product yields and the consumption of glycerol.

This design could be applied in a more extensive screening, which might provide useful genetic backgrounds for the production of H_2_ and ethanol and will help to understand further the physiology of glycerol uptake under anaerobic conditions.

## Results

### Selection of mutants for analysis from the KEIO and YALE collections

In this study, 150 isogenic *E. coli* single knockout mutant strains were cultured under the experimental conditions described in Additional file 1: Figure S1. Each mutant strain was tested in triplicate in every set of experiments and a triplicate of the wild type strain was also analysed as a quality control (QC). The pre-selection of the mutants for analysis was based mainly on genes related to the central carbon metabolism pathways such as glycolysis (8 strains), TCA cycle (20 strains), pentose phosphate pathway (PPP) (13 strains) and intermediate metabolism pathways such as amino acid (20 strains), nucleotide (13 strains) and lipid (13 strains) pathways, although genes involved in other cellular functions were also studied. In this pre-selection some single mutant strains related to the synthesis of hydrogen and ethanol and the assimilation of glycerol were also included (13 strains) and used as experimental controls, since mutants of the same genes have previously been reported to produce higher or lower values for the target products. All of the selected knockout genes are listed in Additional file 2: Table S1.

### Ethanol and H_2_ productions and glycerol consumption in the experimental control strains

**Figure 1:**
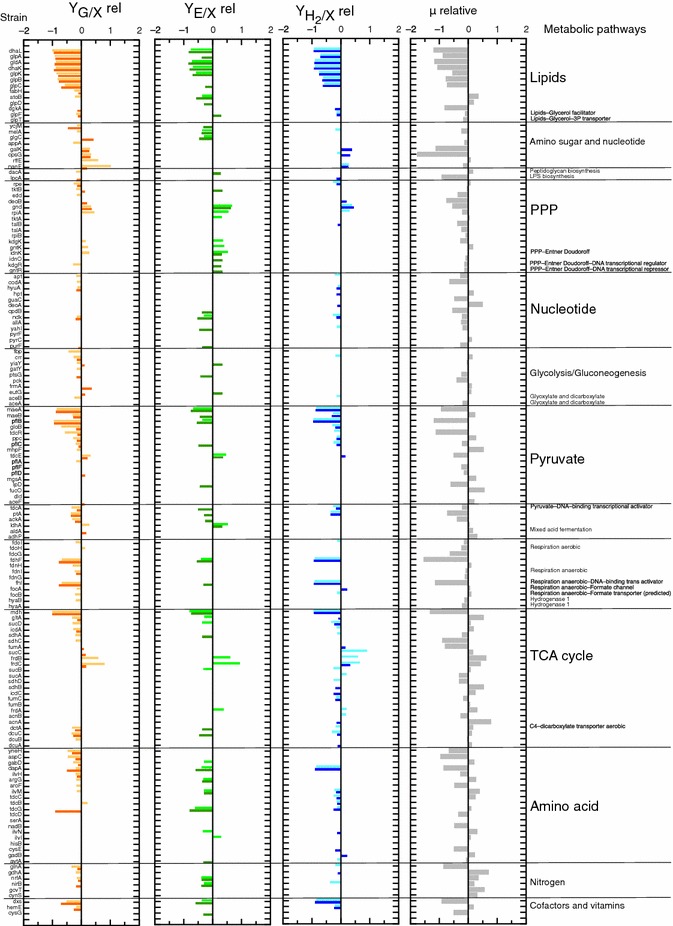
Bar charts showing relative values of the parameters evaluated respect to that of the wild type. Specific glycerol consumed (mmol/g CDW, Y_G/X_ rel) in *orange*; specific ethanol production (mmol ethanol/g CDW, Y_E/X_ rel) in *green*; specific hydrogen production (mmol hydrogen/g CDW, Y_H2/X_ rel) in *blue* and growth rates (*µ* relative) in *grey*. Statistically significant *P* < 0.05 for Y_H2/X_ and Y_G/X_ parameters and 0.01 for Y_E/X_ was used (0 denotes the wild type values). The *clear colours* represent the relative values at 22 h and the *dark colours* at 46 h. In the *left-hand* column are listed the mutant strains assayed in this work and in the *right-hand* column the metabolic pathways in which each defective mutant strain is involved. *LPS* lipopolysaccharide, *PPP* pentose phosphate pathway, *TCA* tricarboxylic acid.

An overview of the experimental control strain values for the target products (Figure 1) showed that the experimental design proposed in this work was appropriate. Thus, the strains defective in enzymes involved in ethanol and H_2_ synthesis and glycerol consumption—that have previously been reported to produce lower yields—namely formate hydrogenolyase (Fhl) [37], pyruvate formate lyase (PflB) [2], glyceraldehyde 3P dehydrogenase (GldABC), dihydroxyacetone kinase (DhaKL) [13], and formate dehydrogenase (FdhF) and formate hydrogenolyase transcriptional factor (FhlA) [13], consistently showed the lowest values for the specific production of the target products as well as the specific consumption of glycerol. On the other hand, the *focA*, *ldhA*, *frdC* [23] *frdA* [14] and *frdB* [38] mutants, which were previously described as efficient H_2_ and/or ethanol producers, showed higher values for these parameters than the wild type reference which is also shown in Figure 1.

### Novel genetic backgrounds suitable for H_2_ and ethanol production and glycerol consumption

In order to determine which of the screened mutants showed statistically significant higher values for Y_E/X_ and/or Y_G/X_ and/or Y_H2/X_ than those of the wild type strain, the non-parametric contrast Mann–Whitney U test was applied to these parameters. From this analysis it was concluded that 45 mutants (including the experimental control strains) showed statistically significant higher values for any of the target products (Additional file 3: Table S3) and all of them showed values for growth rate (µ) > 0 (Additional file 4: Figure S2).

**Figure 2:**
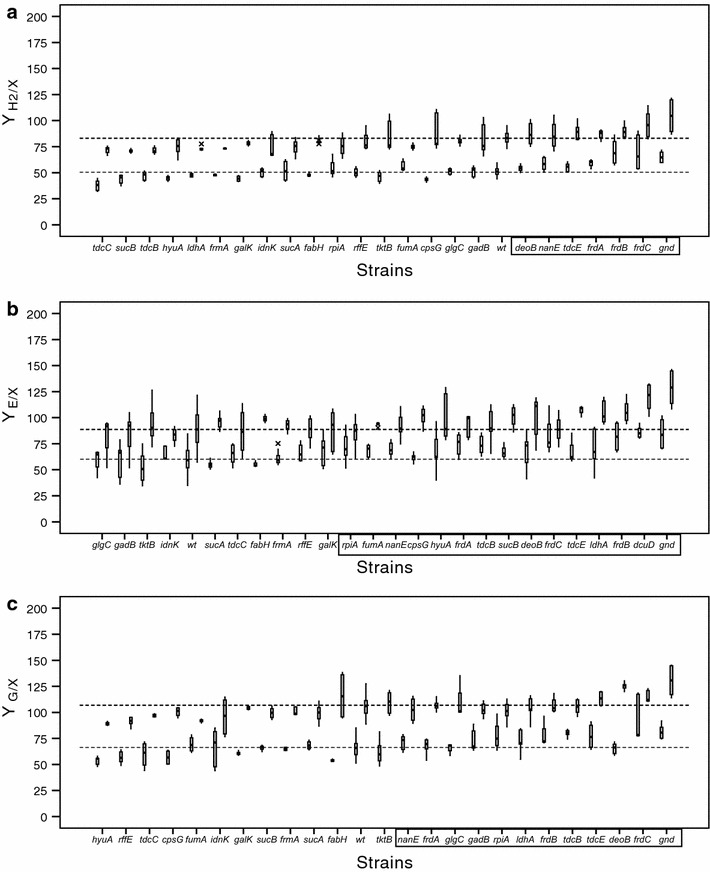
Box plots of parameters evaluated in the selected mutant and wild type strains. Specific hydrogen production (mmol hydrogen/g CDW, Y_H2/X_) (**a**), specific ethanol production (mmol ethanol/g CDW, Y_E/X_) (**b**) and specific glycerol consumption (mmol glycerol consumed/g CDW, Y_G/X_) (**c**). In each graph the *white* and *black boxes* represent the 22 and 46 h interquartile range values respectively and bars the SD. The *dashed lines* in each graph indicate the wild type averages for each parameter at 22 and 46 h. In the X-axis, the strains whose average values are higher with statistical significance in comparison to that of the wild type using a *P* < 0.05 were framed. The wild type data was obtained from at least 75 replicates and the coefficient of variation (CV) was <11% for all parameters, except for ethanol concentration, which was lower than 21%. These results were considered to be suitable to establish a reference for comparison of the mutant strain average values with respect to those obtained for the wild type.

Nevertheless, the number of experiments carried out for each strain was too low (n = 3) to use more robust parametrical analysis such as Student’s *t* test. For this reason, the strains included in the 50th percentile (24 mutants) for each target product (Additional file 3: Table S3) were tested further (n ≥ 6) and the data obtained were analysed in box plots (Figure 2).

**Figure 3:**
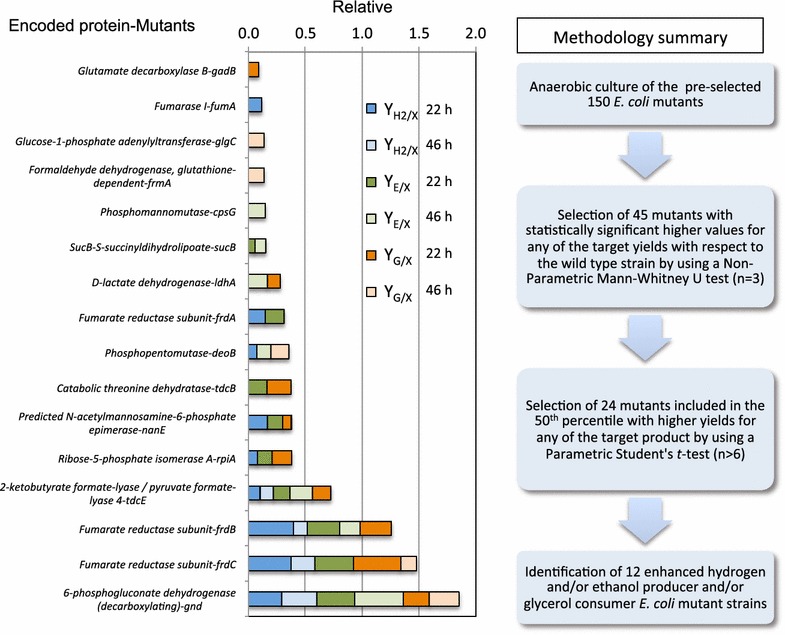
Cumulative bar charts of mutant strains with relative values respect to that of those of the wild type. Specific hydrogen production relative values (Y_H2/X_ rel) coloured in *blue*, specific ethanol production relative values (Y_E/X_ rel) in *green* and specific glycerol consumption in glycerol (Y_G/X_ rel) in *orange* which are significantly higher than the wild type with *P* < 0.05. *Clear colours* represent the 22 h values and the *dark colours* the 46 h ones.

In order to study the interrelation of the three parameters analysed in this work, the Y_H2/X_, Y_E/X_ and Y_G/X_ values for each of these strains and the experimental controls (*frdA*, *frdB*, *frdC* and *ldhA* mutants) were related to the wild type ones and subsequently plotted in the cumulative bar chart shown in Figure 3. These experimental control strains consistently showed similar increased values for at least one of the parameters studied in this work (Additional File 5: Table S4) as previously described in the literature [13, 23, 39, 40]. On the other hand, the remaining 12 mutants (*gnd*, *tdcE*, *rpiAnanE*, *tdcB*, *deoB*, *sucB*, *cpsG*, *frmA*, *glgC*, *fumA* and *gadB*) had not previously been related with the parameters studied in this work. The strains with enhanced values for the three parameters respect to the wild type strain were the *gnd*, *tdcE*, *rpiA*, *nanE* and *deoB* mutants.

The *tdcB* mutant increased the glycerol and ethanol yields. In other cases only one of the analysed parameters was improved: the *sucB* and *cpsG* mutants for ethanol, the *fumA* mutant for hydrogen and the *gadB*, *frmA* and *glgC* mutants for glycerol consumption (Figure 3).

Regarding the values of the cumulative bars, the selected mutants could be separated into three different groups: (1) the *gnd*, *frdC* and *frdB* mutants increased values by more than 100% with respect to the wild type (between 1.12- and 1.43-folds for any of the parameters respect to the wild type); (2) the *tdcE* mutant increased values by more than 50% (from 1.1- up to 1.18-folds respect to the wild type in any of the parameters) and (3) the *rpiA*, *nanE*, *tdcB*, *deoB*, *frdA*, *ldhA*, *sucB*, *cpsG*, *frmA*, *glgC*, *fumA andgadB* mutants showed an increase of <50% than that of the wild type (Figure 3). Consequently, the *gnd* mutant (1.43- and 1.31-folds in ethanol and hydrogen production respectively at 46 h) and *tdcE* mutant (1.19-folds at 46 h and 1.12-folds at 22 h in ethanol and hydrogen production respectively) were selected as novel potential genetic backgrounds for further studies.

### Construction of multiple mutant strains by knocking out the *ldhA*, *gnd*, *frdBC* and *tdcE* genes

One of the strategies commonly used in *E. coli* to improve target product yields is to combine multiple mutations that may help to the redirection of carbon flux towards the synthesis of the desired products. To this aim, the following multiple mutants were constructed; *ldhAgnd*::kan, *ldhAgndfrdBC*::kan and *ldhAgndfrdBCtdcE*::kan denoted in this work as M2, M4 and M5 respectively. On the other hand, another variable that can be considered is the pH condition. In this sense, the same culture medium but at pH 7.5 was analysed as one of the possible variables that could increase the glycerol consumption [14, 22] in the multiple mutant strains. With this purpose, the specific productions for ethanol and hydrogen and glycerol consumption were previously evaluated in the wild type strain up to 94 h (Additional file 6: Figure S3). In these analysis, although specific hydrogen production (Y_H2/X_) expressed in mmol of H_2_/g CDW values did not show significant differences between at both pH (Additional file 6: Figure S3A), the specific ethanol production (Y_E/X_) in mmol of ethanol/g CDW and glycerol consumption (Y_G/X_) in mmol of glycerol consumed/g CDW, were significantly higher at pH 7.5 than those obtained at pH 6.25 from 22 h on (Additional file 6: Figure S3B and C). Therefore the assays with the multiple M2, M4 and M5, the single *gnd* and *tdcE* mutants and wild type strains, were conducted at pH 7.5.

**Figure 4:**
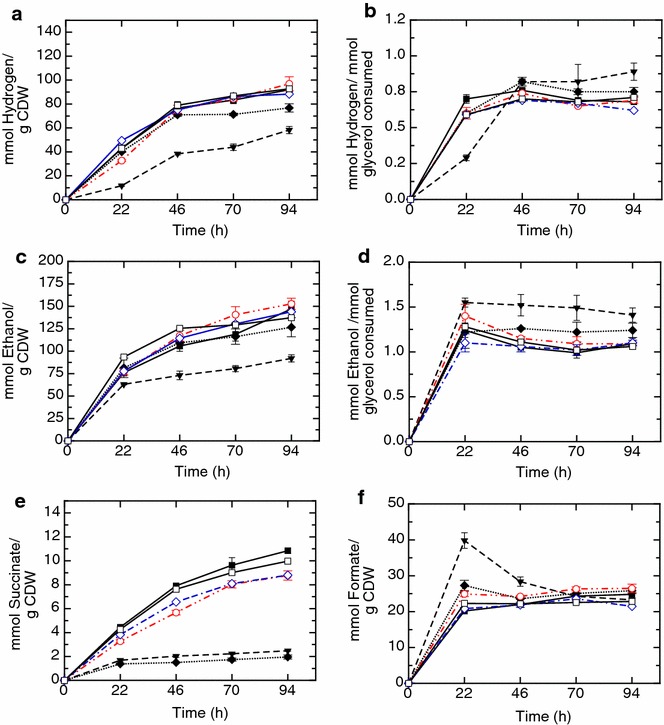
Scatter plots of mean and SD of parameters evaluated in single mutant, multiple mutant and wild type strains. Specific hydrogen production, Y_H2/X_ (**a**); hydrogen molar yield (**b**); specific ethanol production, Y_E/X_ (**c**); ethanol molar yield (**d**); specific succinate efflux, Y_S/X_ (**e**); and specific formate efflux, Y_F/X_ (**f**); graphed up to 94 h in the following mutant and wild type strains: *ldhAgnd*::kan (M2) (*filled square*); *ldhAgndfrdBC*::kan (M4) (*filled diamonds*); *ldhAgndfrdBCtdcE*::kan (M5) (*filled inverted triangle*); *gnd* mutant (*open circle*); *tdcE* mutant (*open square*) and wild type strain (*open diamond*). Time points evaluated were 22, 46, 70 and 94 h of experiment.

Although the Y_H2/X_ and Y_E/X_ in M4 and M5 at 94 h were significantly lower (0.9-folds for both parameters respect to the wild type) than that of the *gnd*, *tdcE* single mutants and for the wild type strain (Figure 4a, c). The molar yields of hydrogen—expressed in mmol of product per mmol of glycerol consumed—was higher for M2 1.07-fold at 22 h and for M5 1.22-fold at 70 h and 1.33-fold at 94 h respect to that of the wild type strain and were also higher than those obtained with M4, *gnd*, and *tdcE* mutants (Figure 4b). Regarding to ethanol molar yields it was found that a value for the wild type strain showed a maximum value of 1.1, which is higher than the theoretical value of 1. This effect can be explained by the fact that the culture medium used in this work is not a minimal medium and, together with glycerol, includes peptone, which can be used by the cells as C source for ethanol production. In the case of M5, ethanol molar yield values were enhanced 1.41-fold from 22 h up to 94 h respect to the wild type and they were also higher than those obtained respect to the other analysed mutant strains (Figure 4d). On the other hand, M5 showed a higher standard deviation (SD) at 70 and 94 h for hydrogen yields and at 46, 70 and 94 h for ethanol yields compared to those obtained at 22 h (Figure 4b, d). This relatively high SD is probably due to a biological variability of this mutant strain in the glycerol consumption since specific production for hydrogen and ethanol (Figure 4a, c) showed very low standard deviation.

In order to better understand the redirection of the carbon rewiring, formate—a precursor of hydrogen synthesis—and succinate—the final product of TCA reductive branch—were analysed in the culture medium. These products are actively exported out from the cell in anaerobic conditions. As can be expected, succinate efflux (Y_S/X_) (mmol/g CDW) was significantly lower for M4 and M5 0.22- and 0.28-fold respectively compare to that of the wild type at 94 h, which are defective in the fumarate reductase enzyme. On the other hand, the M2 showed higher values, 1.23-fold respect to those of the *gnd* mutant and wild type strain at 94 h (Figure 4e). In the case of extracellular formate (Y_F/X_) expressed in mmol/g CDW, the M5 strain showed significantly higher values, up to twofold, at 22 h than those obtained for the M2, and wild type strains and 1.5-fold respect to M4, *tdcE* and *gnd* mutants. These values gradually decreased and eventually reached similar values to that of the *gnd* mutant at 94 h (Figure 4f).

## Discussion

**Figure 5:**
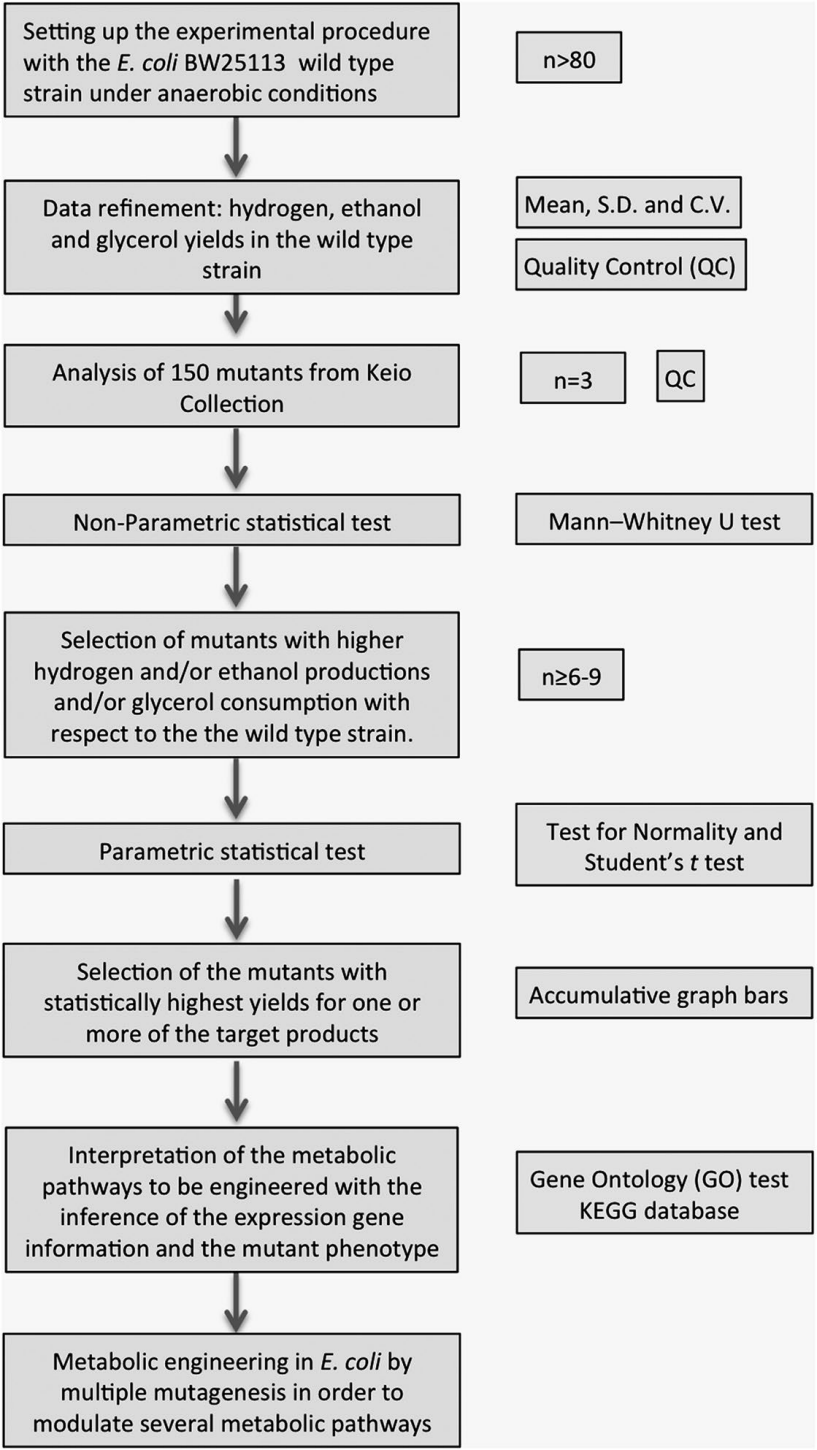
Methodology for a high throughput screening of *E. coli* mutant strains.

The recent development of two knockout *E. coli* collections—the Keio Collection and Yale University CGSC Stock Center [41, 42]—has allowed the systematic search of phenotypes in diverse conditions. Recent advances in high-throughput omic technologies have led to the possibility of deciphering an organism’s genotype-to-phenotype relationships [43]. However, obtaining useful biological knowledge from a single type of omic data—for example, DNA microarray only—is not an easy task. In this work we propose a design for a high-throughput methodology for the systematic analysis of *E. coli* knockout strains for the study of hydrogen and ethanol synthesis and glycerol consumption in order to identify novel *E. coli* phenotypes with enhanced yields for these parameters (Figure 5). In order to test this design, a preliminary screening of 150 single mutants (n = 3) from collection strains (Additional file 2: Table S1) was carried out in this work. This methodological design was considered suitable for our purpose due to its reproducibility. In order to validate the experimental procedure, several mutants used as experimental controls (Figure 1), consistently showed statistically significant differences with respect to the reference strain for the same parameters, which validated this methodology. In addition to the control strains, 45 mutants showed statistically significant enhanced parameters (non-parametric test) for one or more of the target products (Figure 1). Those mutants in the 50th percentile of these 45 strains, for any of the parameters evaluated, were selected for further analysis (Additional file 3: Table S3). These 19 selected mutants, together with the experimental control strains (*ldhA*, *frdA*, *frdB* and *frdC* mutants), were further tested (n ≥ 6) by the parametrical *t* test. In this new analysis the experimental control strains consistently showed increased Y_H/X_, Y_E/X_ and Y_G/X_ (Figure 2) and also confirmed that 12 out of the 19 mutant strains showed an enhanced ethanol and/or hydrogen production and/or glycerol consumption (*gnd*, *tdcE*, *rpiAnanE*, *tdcB*, *deoB*, *sucB*, *cpsG*, *frmA*, *glgC*, *fumA* and *gadB*). These mutated genes are mainly related to the metabolism of amino acids, PPP, TCA, gluconeogenesis, amino sugar and nucleotides.

It is remarkable that 2 out of the 12 selected mutant strains were defective in enzymes involved in the PPP, including the *gnd* mutant—defective for the 6-phosphogluconate dehydrogenase enzyme—for which Y_H/X_, Y_E/X_ and Y_G/X_ parameters were significantly enhanced at the two times *post inoculum* studied in this work. A possible explanation for this phenotype may be that the lack of activity of this enzyme can increase the pool of 6-PG, which would lead to an increase in glyceraldehyde 3-P (G3P) and phosphoenolpyruvate (PEP) by the Entner–Doudoroff pathway, which feeds the ‘bottom half’ of glycolysis [44]. On the other hand, the *rpiA* mutant (defective in ribulose-5P-isomerase) also increased the three parameters analysed in this work, albeit to a lesser extent than the *gnd* mutant. The enzyme codified by this gene is involved in the interconversion of ribose-5P to ribulose-5P. The lack of this enzyme could also have a similar effect to that of the *gnd* mutant, i.e., the increase of glyceraldehyde 3-phosphate (GA3P) and fructose-6P from pentose sources. Another mutant that is related to the metabolism of pentoses was also found in the 12 selected mutants, namely deoB phosphopentomutase mutant, which showed a similar phenotype to that of the *rpiA* mutant. This enzyme is involved in the catabolism of nucleotides and deoxynucleotides and promotes the interconversion of ribose-1P or deoxyribose-1P to their corresponding 5P form. It can therefore be concluded from these results that the reorganization of the metabolism through the blocking of several enzymes involving the PPP is a promising possibility for the rewiring of the metabolism in the use of glycerol as the main C source towards pyruvate and, subsequently synthesising acetyl-CoA and formate which are transformed respectively into ethanol and hydrogen.

In this screening 39 mutants that involve the nitrogen metabolites were also tested. From these mutants only the *tdcE* (2-ketobutyrate formate-lyase/pyruvate formate lyase) and the *tdcB* (threonine dehydratase) mutants, both of which codify enzymes involved in threonine degradation [45], showed enhanced hydrogen yield and glycerol consumption and the *tdcE* mutant also showed a higher ethanol yield. In this regard, the mutation of any of these enzymes may increase the acetyl-CoA pool and, consequently, the ethanol production. Although not all of the enzymes related to amino acid metabolism were tested, it is significant that two of the selected mutants were related to threonine catabolism. Nevertheless, more in-depth studies are needed to investigate the possibility of rewiring the amino acid metabolism and the rest of the mutants (*nanE*, *deoB*, *sucB*, *cpsG*, *frmA*, *glgC*, *fumA*, *gadB*) found in this work.

In order to gain a deeper understanding of which biological functions could be depleted to avoid redundant or deleterious mutations, with the ultimate aim of constructing a multiple mutant strain, these genes were analysed in the Gene Ontology (GO) database [46]. Although several of the GO matches proved to be redundant, the most significant biological processes that were affected were the fermentation, energy derivation by oxidation of organic compounds, generation or precursor metabolites and energy, cell projection, generation of precursor metabolites and energy, oxidation–reduction process and flagella motility among others (Table 1). The results of this analysis seem to indicate that the cells try to rewire the metabolic process to maintain the energy balance, which is translated to the alteration in the synthesis of precursors for the anabolic metabolic processes that cause a decrease in the cell division rate, as can be observed by the relative µ values for the mutants studied in this work (Additional file 4: Figure S2). In addition, the oxidation–reduction process involving the NAD(P)H/NAD(P)^+^ balance within the cell is fundamental in the equilibrium of glycolysis and fermentation pathways in order to synthesis ATP by level-substrate phosphorylation and also in thousands of energetic-dependent reactions.

**Table 1:** Gene-Ontology (GO) database search in EcoCyc of the knockout mutants selected in based on the statistically significant results of the parameters evaluated shown in Figure 3

Gene-Ontology-terms	p-values^a^	Matches (mutant strains)
Fermentation	2.22E−08	*ldhA/*/*“frdB”//“frmA”//“frdA”//“frdC”*
Generation of precursor metabolites and energy	1.58E−07	*frdB//“tdcE”//“frdA”//“frdC”//“glgC”//“ldhA”//“frmA”//“fumA”*
Energy derivation by oxidation of organic compounds	5.45E−07	*frdB//“tdcE”//“frdA”//“frdC”//“glgC”//“ldhA”//“frmA”*
Aspartate family amino acid catabolic process	2.02E−05	*sucB//“tdcB”//“tdcE”*
Bacterial-type flagellum assembly	2.57E−05	*frdA//“frdB”//“frdC”*
Ethanol metabolic process	5.57E−05	*ldhA//“frmA”*
Oxidation–reduction process	6.95E−05	*gnd//“rpiA”//“frdB”//“tdcE”//“frdA”//“frdC”//“glgC”//“ldhA”//“frmA”*
Single-organism metabolic process	9.44E−05	*rpiA//“gnd”//“deoB”//“gadB”//“tdcB”//“frdB”//“tdcE”//“frdA”//“frdC”//“glgC”//“frmA”//“cpsG”//“ldhA”//“sucB”//“nanE”*
Single-organism catabolic process	1.52E−04	*ldhA//“gnd”//“rpiA”//“deoB”//“tdcB”//“sucB”//“tdcE”//“nanE”//“frmA”*
Tricarboxylic acid cycle	1.57E−04	*fumA//“frdB”//“sucB”*
l-threonine catabolic process to propionate	3.86E−04	*tdcB//“tdcE”*
Anaerobic respiration	4.10E−04	*frdB//“tdcE”//“frdA”//“frdC”*
Organic substance catabolic process	6.34E−04	*frmA//“tdcB”//“sucB”//“tdcE”//“ldhA”//“gnd”//“rpiA”//“nanE”//“deoB”*
Glucose catabolic process	6.49E−04	*ldhA//“gnd”//“rpiA”*
Cell motility	8.04E−04	*frdA//“frdB”//“frdC”*
Carbohydrate metabolic process	8.48E−04	*rpiA//“gnd”//“ldhA”//“glgC”//“cpsG”//“nanE”//“tdcE”*
Small molecule metabolic process	8.52E−04	*frmA//“cpsG”//“deoB”//“gnd”//“rpiA”//“ldhA”//“gadB”//“tdcB”//“sucB”//“tdcE”//“nanE”*
Cellular metabolic process	8.65E−04	*deoB//“frdB”//“tdcE”//“frdA”//“frdC”//“frmA”//“gnd”//“rpiA”//“cpsG”//“glgC”//“ldhA”//“gadB”//“tdcB”//“nanE”//“sucB”//“fumA”*
Cellular respiration	9.59E−04	*frdB//“tdcE”//“frdA”//“frdC”*

^a^The matches were found using a *P* < 0.001 which are denoted as scientific notation (E).

Once established the main metabolic pathways, in which these mutants were involved, the *gnd* and *tdcE* mutants were selected for further analysis because they showed the higher values in all of the parameters evaluated respect to the wild type strain. One of the strategies commonly used to enhance the efficiency of the biotransformation processes carried out by *E. coli* is the redirection of C-flux by multiple mutations. In this work the mutation of *gnd* gene (defective in the synthesis of d-Ribulose 5-P) was combined with the deletion of lactate synthesis (*ldhAgnd::kan*, M2), the succinate synthesis (*ldhAgndfrdBC*::kan, M4) and the threonine degradation (*ldhAgndfrdBCtdcE*::kan, M5). These mutant strains together with the reference strain were also evaluated at pH 7.5 due to it has been previously described that glycerol metabolization is favored at this pH [22]. In the case of M4 and M5 mutants, the blockage of succinate synthesis was observed because in both strains the succinate efflux was substantially decreased (Figure 4e) as can be expected from the mutation of *frdBC* genes [7, 47]. The hydrogen and ethanol molar yields obtained from these engineered strains, indicates that metabolism of C source is shunt towards formate, in the case of *gnd* and *tdcE* single mutants, M4 and specially M5 mutant in which formate is increased twofold respect wild type strain and 1.5-fold respect to *gnd* mutant (Figure 4f).

This effect correlated with an enhanced ethanol molar yield in the M5 mutant (1.41-fold respect to that of the wild type) (Figure 4d). This improvement is higher than that obtained in the septuplet mutant (BW25113*frdC ldhA fdnG ppc narG mgsA*, *hycA*) reported by Tran et al. [23] (0.67-fold respect to the wild type at 24 h). The only difference between M4 and M5 strains is the deletion of TdcE enzyme gene, which is involved in catabolism of threonine into propanoate [45]. This deletion in M5 may provoke a significant metabolic redirection—through unknown metabolic regulation mechanisms—towards pyruvate and subsequently to formate—which is exported out the cell- and acetyl-CoA that is converted more efficiently into ethanol (Figure 4d). The excess of formate is exported out the cell due to accumulation of this molecule in the cell to toxic levels, although after 22 h could be imported [31, 48] and then converted to hydrogen (Figure 4a). In fact, the M5 strain growth was significantly depleted after 22 h (Additional file 7: Figure S4) and specific productions were very low respect to all the analysed strains (Figure 4a, c). In this mutant the higher molar yield measured for hydrogen and ethanol could mean that glycerol consumption was lower, although the metabolization of this product is more efficient in M5 mutant than in the wild type. In this sense M5 mutant could be an interesting genetic background for further studies in which the excess of formate may be converted into hydrogen.

## Conclusion

In conclusion, the systematic analysis of the target products in *E. coli* mutant strains proposed in this work is a feasible methodology to identify novel suitable genetic backgrounds to enhance the synthesis of hydrogen and/or ethanol in cells cultured in a glycerol-based medium. In this work we identified several mutants (chiefly *gnd*, and the *tdcE*) that could be combined in multiple mutant strains in order to enhance the yields of the desired products by metabolic engineering. This kind of studies can also help to understand the metabolic rewiring to reveal the pathways that are most susceptible to genetic modification, which could in turn facilitate the design of more efficient strategies to engineer *E. coli* strains.

## Methods

### Bacterial strains and chemicals

The strain BW25113 was used as the wild type strain in this work. Isogenic single-gene knock out derivatives [48] were obtained from the National Bioresource Project, Keio Collection (NIG, Japan) and from the Coli Genetic Stock Center (CGSC) (Yale University, USA) [41] and they are listed in Additional file 2: Table S1.

Molecular and functional information for the metabolic pathways used in this work was compiled from the Kyoto Encyclopaedia of Genes and Genomes (KEGG) (http://www.genome.jp/kegg/) [49] and EcoCyc (http://ecocyc.org/) [50].

Kanamycin (Kan) was purchased from Gibco™ (Invitrogen, UK) and was used for pre-culturing the isogenic knockouts with chromosomal kan resistance markers (Kan^R^) at a concentration of 50 µg mL^−1^. The chemicals used for the culture media were as follows: peptone, yeast extracts, agar–agar were obtained from Panreac (PANREAC QUIMICA S.A., Spain) and KH_2_PO_4_, Na_2_HPO_4_ (extra pure), Na_2_SO_4_, NaCl, MgSO_4_^∙^7H_2_O and glycerol (food grade 99% extra pure) were obtained from Scharlau (Scharlab S.L., Spain).

### Culture conditions

*Escherichia coli* knock out strains listed in Additional file 2: Table S1 were initially streaked from −80°C glycerol stocks on LB agar plates containing Kan. Knock out mutations were checked by PCR [50]. Cultures for all of the experiments were incubated in an orbital incubator shaker at 200 rpm and 37°C.

For the screening of 150 single mutant strains, a fresh single colony was inoculated in 2 mL Luria–Bertani (LB) medium supplemented with Kan and the colony was cultured overnight. This aerobic pre-culture was used to inoculate the bacteria under microaerobic conditions in LB-glycerol medium [11] in 50 mL Falcom tubes (VWR International Eurolab S.L., Spain). These cells were centrifuged at 4,900×*g* for 15 min at 4°C (Sigma 2K15, Laborzentrifugen GmbH, Germany). Inside an anaerobic glove box, previously purged with Ar to diminish the oxygen level to below 1%, the pellet obtained was suspended in approximately 40 mL glycerol-based medium [11], (KH_2_PO_4_, 7.19 g L^−1^; Na_2_HPO_4_, 1.98 g L^−1^; Na_2_SO_4_, 0.0806 g L^−1^; NaCl, 0.0152 g L^−1^; MgSO_4_^∙^7H_2_O, 0.031 g L^−1^; glycerol 10 g L^−1^ (109 mM) and peptone 4.25 g L^−1^, pH 6.25) previously sparged with Ar for 5 min, in order to obtain an OD_600nm_ of 0.83 ± 0.025. These cells were poured into 12 mL crimp-top vials and sealed with a butyl rubber septum and aluminium caps. Both LB-glycerol and glycerol-based medium were previously sparged with argon (Ar) gas (99.9%) for 5 min to ensure that they were completely deprived of O_2_. The wild type and the *gnd* and the multiple mutant strains were also assayed in the experimental conditions described previously except for the glycerol-based medium pH, which was adjusted at pH 7.5 by using the following salt buffer concentrations: KH_2_PO_4_, 1.78 g L^−1^ and Na_2_HPO_4_, 7.65 g L^−1^. Triplicates of each *E. coli* strains were incubated for 22 and 46 h.

### Construction of knock out strains

The multiple mutants were constructed using the *ldhA*::kan single mutant as the parental strain following the homologous recombination method described by Datsenko and Wanner [51]. Firstly, the Kan resistance marker inserted in the *ldhA* mutant strain (Δ*ldhA*::kan) was removed after transformation with the pCP20 plasmid. Clones were selected by replica plating in LB agar plates supplemented with Cm or Kan. For plasmid curing, several clones were randomly selected; replica plated in LB agar plates with no antibiotic and incubated at 42°C. For the multiple strain constructions, *gnd*, *frdBC* and *tdcE* genes, pairs of primers were designed in order to have short 5′ (H1) or 3′ (H2) homology sequences of the target genes (in capital letters) that were flanking P4 or P1 priming sequences of the pKD13 vector (in lower case letters) (Additional file 8: Table S2). Using these primers and the pKD13 vector as the template, PCR products were performed containing the Kan^R^ gene and flanked by FLP recognition targets (FRTs). PCR was carried out with Velocity™ DNA polymerase (Bioline Reagents Ltd., London, UK). The PCR-generated products were transformed by electroporation in the strains previously transformed with the pKD46 plasmid. The transformants were then selected in LB agar plates supplemented with Kan. Gene disruptions of the generated mutant strains; were confirmed by PCR amplification by using external and internal primers (Additional file 8: Table S2).

All DNA manipulations were performed according to standard methods [52, 53], plasmid isolation was achieved using the NucleoSpin^®^ Plasmid Kit (Macherey–Nagel, Düren, Germany GmbH & Co.) and PCR Clean-up was performed with the QIAquick PCR Purification Kit (QUIAGEN, Hilden, Germany).

### Analytical techniques and methods

The volume of gas generated (H_2_ and CO_2_) in the headspace was measured by inserting a needle, which was connected to a water column manometer, into the rubber septum. Hydrogen quantification in the headspace was measured by injecting 100 µL aliquots into a Bruker 450-Gas Chromatograph (GC) equipped with a Poraplot Q Plot FS 25 × 53 column and a thermal conductivity detector (TCD) (Bruker Daltonik GmbH, Fahrenheitstr, Germany). The injector and detector were maintained at 250 and 150°C, respectively and the Ar carrier gas flow rate was maintained at 10 mL min^−1^.

Cell growth was estimated by measuring OD_600 nm_ (1 OD = 0.31 g of cell dry weight (CDW)/L) according to standard procedures [54] on a Spectroquant^®^ Pharo 100 spectrophotometer (© Merck KGaA, Darmstadt, Germany).

Glycerol, ethanol, succinate and formate efflux, were measured from the supernatant of the samples, filtered through 0.22 µm nylon filters, and quantified by HPLC as previously described [11].

### Calculation of parameters and statistical analysis

The raw data for H_2_ (%), concentration of ethanol and glycerol consumed (g L^−1^) and the volume of gas generated (mL) were analysed and were used to calculate the following parameters for the wild type and mutant strains: specific hydrogen (Y_H2/X_) and ethanol productions (Y_E/X_), specific glycerol consumption (Y_G/X_) values referred to the biomass (X) (g of CDW). For the multiple mutant, *gnd* and *tdcE* single mutants and wild type strains, succinate (Y_S/X_) and formate efflux (Y_F/X_) were also calculated and referred to the biomass.

For each parameter the average (m), standard deviation (SD) and the coefficient of variation (CV) were calculated using at least three biological replicates for the mutant strains and at least 75 replicates for the wild type strain. For the experiments with the pre-selected mutant, the statistical analysis for each mutant parameter was considered to be significantly different based on the non-parametric contrast of the Mann–Whitney U test. The *P* < 0.01 for Y_E/X_ and <0.05 for Y_H2/X_, CDW, and Y_G/X_ parameters were used. The statistically significant values of the Y_H2/X_, Y_E/X_, Y_G/X_, and µ parameters (Y rel.) were used to relativize the mutant values with the wild type ones (Y_mut_–Y_wt_/Y_wt_) (wild type denoted as 0). The molar yields for H_2_ and ethanol (mmol product/mmol glycerol consumed) were also calculated in the multiple mutant, *gnd* and *tdcE* single mutants, and wild type strains.

For mutant selection on the basis of the best parameter (Y_H2/X_, Y_G/X_ and Y_E/X_ results) obtained at 22 and/or 46 h, the distribution of continuous variables was evaluated by the Shapiro–Wilk’s normality test and Levene’s test for homogeneity of variances was employed to inform the choice of the appropriate statistical test. As conditions for the application of parametric tests, Student’s *t* test was used to evaluate the statistical significance of differences the parameters between the groups. IBM^®^ SPSS^®^ Statistics 20 software was used for statistical analysis.

Gene Ontology (GO) database information on gene expression was used to provide additional information about the selected mutant phenotypes (Table 1). GO is a database of standardized biological terms used to annotate gene products and it comprises several thousand terms divided into three branches: molecular function, biological process and cellular component. This analysis of putative biological processes was applied to the selected mutants and the assignment of a biological process was restricted to a *P* < 0.001 in the Fisher’s test was used in this database.

## Additional files

Additional file 1:
**Figure S1**. In the screening carried out in this work, the culture conditions were first normalized and the procedures for the measurement of the ethanol and hydrogen production and the glycerol consumption were established by a gradual adaptation of the bacteria to anaerobic growth and the use of glycerol as the carbon source. This adaptation process is summarized in four steps; the overnight aerobic pre-culture grown in LB medium **(A)**, 1% of microaerobic inoculum **(E)** cultured in LB-glycerol containing a negligible oxygen concentration culture up to 4 h **(B)**; the anaerobic inoculum, in which the LB-glycerol medium is removed and replaced by the glycerol-based medium **(C)**; and the anaerobic mini-reactors **(D)**; with 60% headspace was chosen for higher hydrogen production and two time points at 22 and 46 h post-inoculum due to were representative of the LP and SP for the analysis of the target products **(F)**. Additional file 2:
**Table S1.** List of *E. coli* knock out mutant strains purchased from Keio and Yale collections. Additional file 3:
**Table S3.** *E. coli* mutant strains with significantly higher values of specific hydrogen production (Y_H2/X_) in blue, specific ethanol production (Y_E/X_) in green and specific glycerol consumption (Y_G/X_) in orange with respect to the wild type values. Intensity of colours indicates higher relative values. The 50th percentile of the values are: 14% and 13% for the Y_H2/X_ at 22 and 46 h, respectively, 47% and 33% for the Y_E/X_ at 22 and 46 h respectively, and 32% and 20% for the Y_G/X_ at 22 and 46 h respectively. The mutant strains selected according to the 50th percentile are marked in bold Additional file 4:
**Figure S2.** Growth rates (μ) calculated for the mutants and wild type strain (in frame) ordered from top to bottom in ascendant order. Additional file 5:
**Table S4.** Relative hydrogen and ethanol production in *E. coli* mutant strains using different carbon sources and pH. Additional file 6:
**Figure S3.** Scatter plots of mean and SD of specific hydrogen production, Y_H2/X_ **(A)**; specific ethanol production, Y_E/X_ **(B)**; and specific glycerol consumption, Y_G/X_ **(C)** of wild type strain with culture medium at pH 6.25 (*filled inverted triangle*) and pH 7.5 (*open circle*). Additional file 7:
**Figure S4.** Cell dry weight (CDW) curves in g/L of the multiple mutants*: ldhAgnd*::kan (M2) (*filled square*); *ldhAgndfrdBC*::kan (M4) (*filled diamond*)*; ldhAgndfrdBCtdcE*::kan (M5) (*filled inverted triangle*); the single mutants: *gnd* (*open circle*) and *tdcE* (*open square*) and the wild type strain (*open diamond*). Additional file 8:
**Table S2.** The primers from 1 to 6 were used to obtain multiple mutants; *ldhAgnd:kan* (M2), *ldhAgndfrdBC::kan* (M4) and *ldhAgndfrdBCtdcE::kan* (M5). Capital letters indicate the homologous sequences of the target genes and in lower case letters indicate the priming sequences of the pKD13 vector. In the names of the primers, H2P1 indicates the forward primers and H1P4 the reverse primers. Primers from 7 to 15 were used for PCR verification of the deletion of the targeted genes.

## Abbreviations

GLDA: glycerol dehydrogenase
PflB: pyruvate formate lyase
GldABC: glyceraldehyde 3P dehydrogenase
DhaKL: dihydroxyacetone kinase
FdhF: formate dehydrogenase-H
FhlA: formate hydrogenolyase transcriptional factor
PPP: pentose phosphate pathway
TCA: tricarboxilyc acid cycle
GA3P: glyceraldehyde 3-phosphate
PEP: phosphoenolpyruvate
GO: gene ontology
QC: quality control
µ: growth rate
