# Supplementary figures and images for: Identification of enhanced hydrogen and ethanol Escherichia coli producer strains in a glycerol-based medium by screening in single-knock out mutant collections
Source: Microb Cell Fact. 2015 Jun 28;14:93. doi: 10.1186/s12934-015-0285-6 (PMC4485358; doi:10.1186/s12934-015-0285-6)

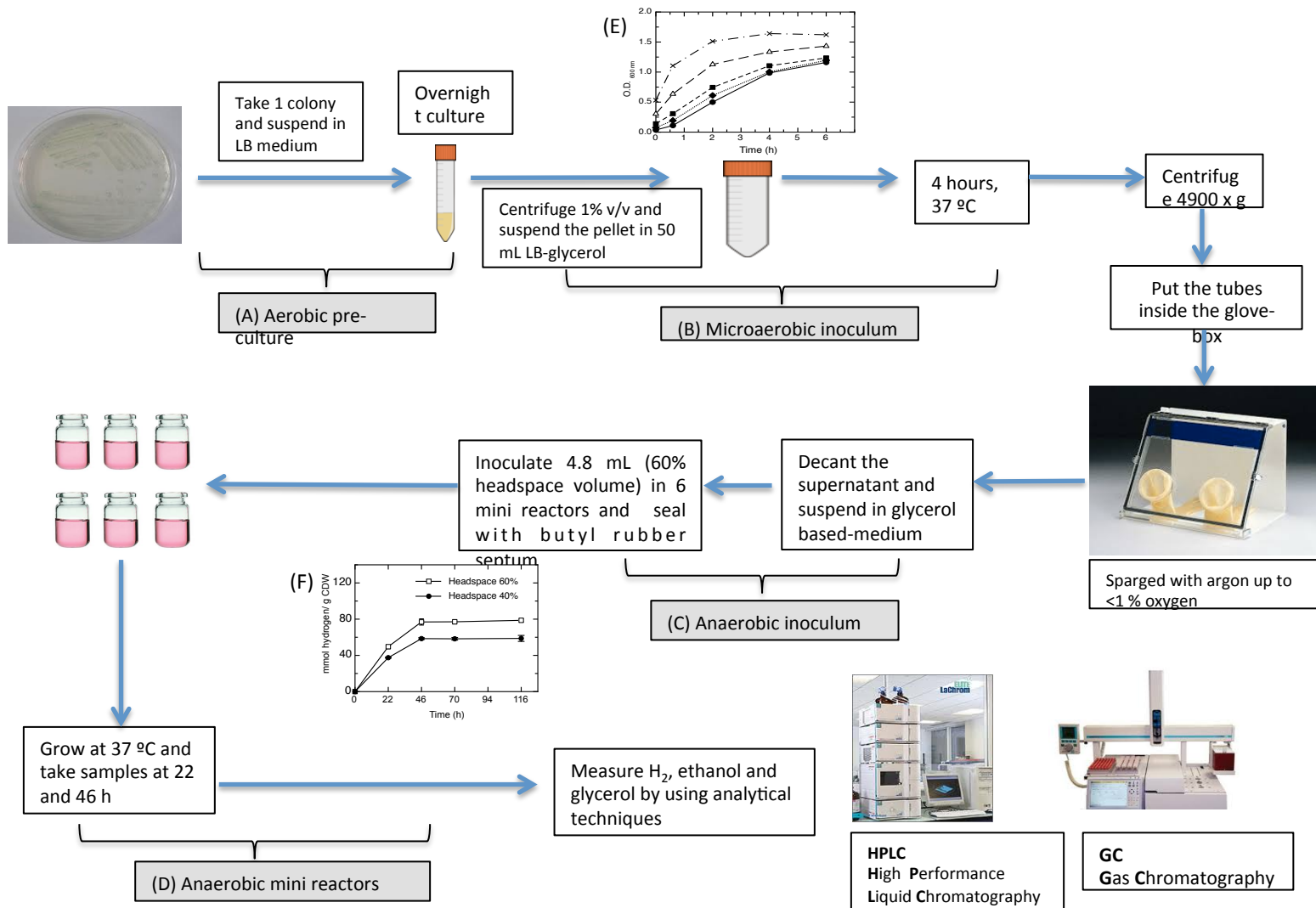

Supplement: Additional file 1: — Figure S1. In the screening carried out in this work, the culture conditions were first normalized and the procedures for the measurement of the ethanol and hydrogen production and the glycerol consumption were established by a gradual adaptation of the bacteria to anaerobic growth and the use of glycerol as the carbon source. This adaptation process is summarized in four steps; the overnight aerobic pre-culture grown in LB medium (A), 1% of microaerobic inoculum (E) cultured in LB-glycerol containing a negligible oxygen concentration culture up to 4 h (B); the anaerobic inoculum, in which the LB-glycerol medium is removed and replaced by the glycerol-based medium (C); and the anaerobic mini-reactors (D); with 60% headspace was chosen for higher hydrogen production and two time points at 22 and 46 h post-inoculum due to were representative of the LP and SP for the analysis of the target products (F). [file 12934_2015_285_MOESM1_ESM.pdf]

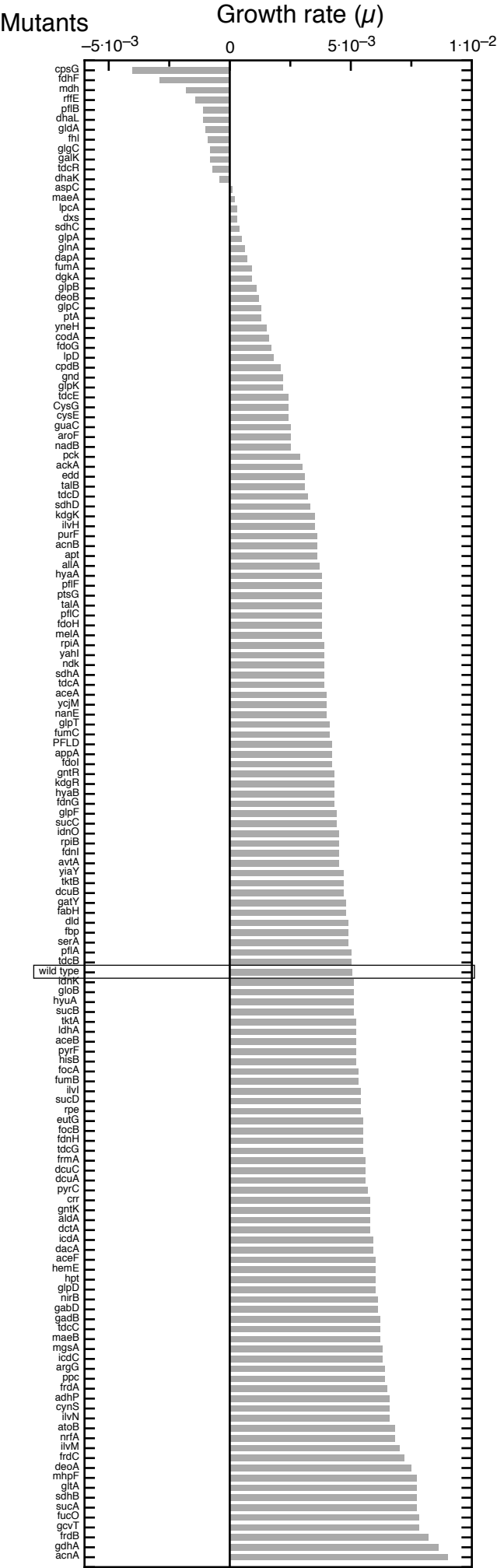

Supplement: Additional file 4: — Figure S2. Growth rates (μ) calculated for the mutants and wild type strain (in frame) ordered from top to bottom in ascendant order. [file 12934_2015_285_MOESM4_ESM.pdf]

**A**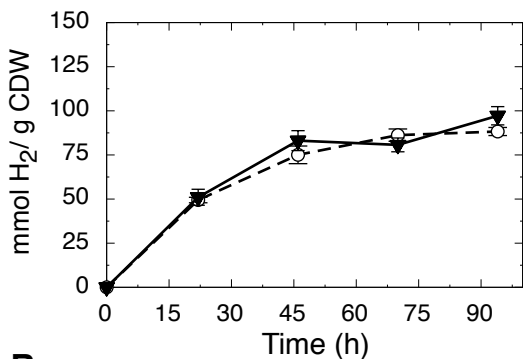**B**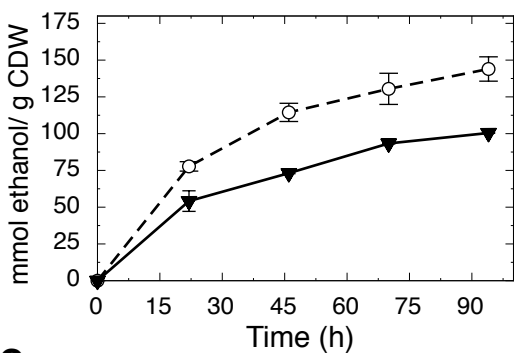**C**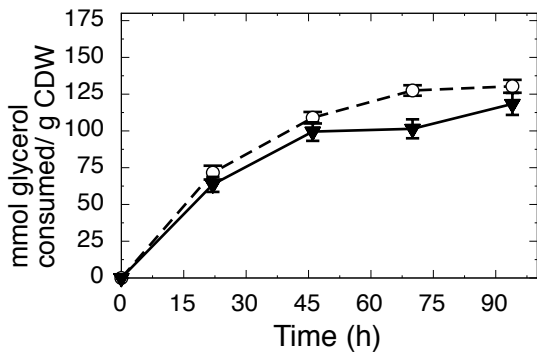

Supplement: Additional file 6: — Figure S3. Scatter plots of mean and SD of specific hydrogen production, YH2/X (A); specific ethanol production, YE/X (B); and specific glycerol consumption, YG/X (C) of wild type strain with culture medium at pH 6.25 (filled inverted triangle) and pH 7.5 (open circle). [file 12934_2015_285_MOESM6_ESM.pdf]

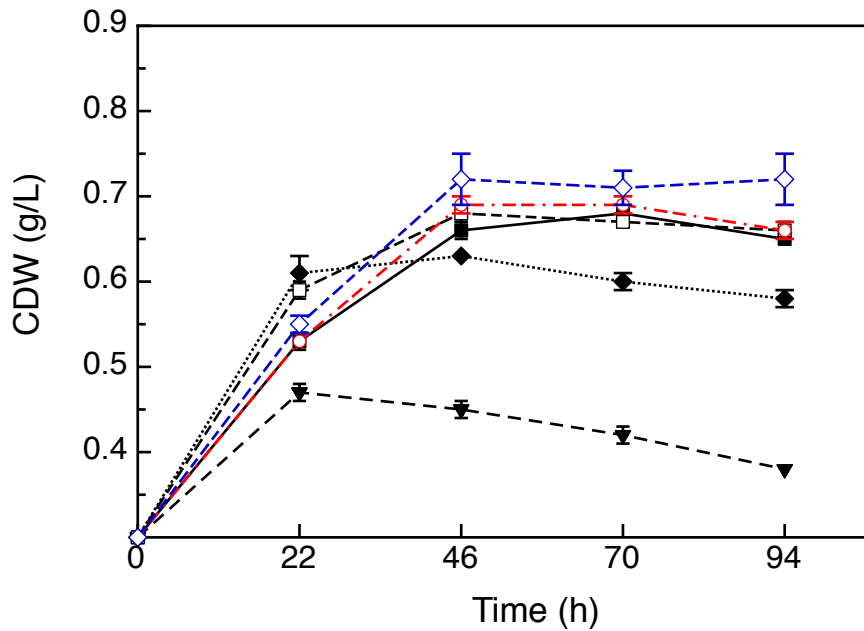

Supplement: Additional file 7: — Figure S4. Cell dry weight (CDW) curves in g/L of the multiple mutants: ldhAgnd::kan (M2) (filled square); ldhAgndfrdBC::kan (M4) (filled diamond); ldhAgndfrdBCtdcE::kan (M5) (filled inverted triangle); the single mutants: gnd (open circle) and tdcE (open square) and the wild type strain (open diamond). [file 12934_2015_285_MOESM7_ESM.pdf]
