# Supplementary material for: Identification of enhanced hydrogen and ethanol Escherichia coli producer strains in a glycerol-based medium by screening in single-knock out mutant collections
Source: Microb Cell Fact. 2015 Jun 28;14:93. doi: 10.1186/s12934-015-0285-6 (PMC4485358; doi:10.1186/s12934-015-0285-6)
Supplement: Additional file 2: — Table S1. List of E. coli knock out mutant strains purchased from Keio and Yale collections. [file 12934_2015_285_MOESM2_ESM.pdf]

Gene knockouts mutants	Accession no. (GeneBank)	Defective gene products
aceA	b4015	Isocitrate lyase monomer
aceB	b4014	Malate synthase A
aceF	b0115	Pyruvate dehydrogenase, dihydrolipoyltransacetylase component E2
ackA	b2296	Acetate kinase
acnA	b1276	Aconitate hydratase 1 (Aconitase 1)
acnB	b0118	Bifunctional aconitate hydratase 2 and 2-methylisocitrate dehydratase (Aconitase 2)
adhP	b1478	Ethanol-active dehydrogenase/acetaldehyde-active reductase
aldA	b1415	Aldehyde dehydrogenase A, NAD-linked
allA	b0505	Ureidoglycolate lyase
appA	b0980	Acid phosphatase
apt	b0469	Adenine phosphoribosyltransferase
argG	b3172	Argininosuccinate synthase
aroF	b2601	2-dehydro-3-deoxyphosphoheptonate aldolase
aspC	b0928	Aspartate aminotransferase, PLP-dependent
atoB	b2224	Acetyl-CoA C-acetyltransferase
avtA	b3572	Valine-pyruvate aminotransferase
codA	b0337	Cytosine deaminase
cpdB	b4213	2',3'-cyclic nucleotide 2'-phosphodiesterase / 3'-nucleotidase
cpsG	b2048	D-mannose 1,6-phosphomutase
crr	b2417	Glucose-specific enzyme IIA component of PTS/ permease
cynS	b0340	Cyanase (cyanate hydrolase)
cysE	b3607	Serine acetyltransferase component of bifunctional CysEK cysteine biosynthesis complex
cysG	b3368	Uroporphyrin III C-methyltransferase (multifunctional)
dacA	b0632	D-alanyl-D-alanine carboxypeptidase
dapA	b2478	4-hydroxy-tetrahydrodipicolinate synthase
dctA	b3528	C4 di-carboxylate transporter aerobic
dcuA	b4138	C4 di-carboxylate transporter anaerobic
dcuB	b4123	C4 di-carboxylate transporter anaerobic
dcuC	b0621	C4 di-carboxylate transporter anaerobic
dcuR	b4124	DcuR-Phosphorylated DNA-binding transcriptional activator
deoA	b4382	Thymidine phosphorylase / uracil phosphorylase
deoB	b4383	Phosphopentomutase
dgkA	b4042	Diacylglycerol kinase
dhaK	b1200	Dihydroxyacetone kinase subunit K
dhaL	b1199	Dihydroxyacetone kinase subunit L
dld	b2133	D-lactate dehydrogenase
dxs	b0420	1-deoxyxylulose-5-phosphate synthase
edd	b1851	Fosfogluconate dehydratase
eutG	b2453	Predicted alcohol dehydrogenase in ethanolamine utilization
fabH	b1091	3-oxo-glutaryl-(ACP)methyl ester synthase; β-ketoacyl-ACP synthase (KASIII)

fbp	b4232	Fructose 1,6 bisphosphatase I
fdhF	b4079	Formate dehydrogenase-H (hydrogenase 3)
fdnG	b1474	Formate dehydrogenase-N, α subunit
fdnH	b1475	Formate dehydrogenase-N, β subunit
fdnI	b1476	Formate dehydrogenase-N, γ subunit
fdoG	b3894	formate dehydrogenase-O, α subunit
fdoH	b3893	formate dehydrogenase-O, β subunit
fdoI	b3892	formate dehydrogenase-O, γ subunit
fhlA	b2731	FhlA-Formate DNA-binding transcriptional activator
focA	b0904	Formate channel
focB	b2492	Formate nitrite transporter (FNT)
frdA	b4154	Fumarate reductase flavoprotein
frdB	b4153	Fumarate reductase iron-sulfur protein
frdC	b4152	Fumarate reductase subunit anchor to inner membrane
frdD	b4152	Fumarate reductase subunit anchor to inner membrane
frmA	b0356	S-(hydroxymethyl)glutathione dehydrogenase / alcohol dehydrogenase
fucO	b2799	L-1,2-propanediol oxidoreductase
fumA	b1612	Fumarase A class I
fumB	b4122	Fumarase B class I
fumC	b1611	Fumarase C class II
gabD	b2661	Succinate-semialdehyde dehydrogenase (NADP+)
gadB	b1493	Glutamate decarboxylase β subunit
galK	b0757	Galactokinase
gatY	b2096	Tagatose-1,6-bisphosphate aldolase 2
gcvT	b2905	Aminomethyltransferase
gdhA	b1761	Glutamate dehydrogenase
gldA	b3945	L-1,2-propanediol dehydrogenase / glycerol dehydrogenase
glgC	b3430	Glucose-1-phosphate adenylyltransferase
glnA	b3870	Glutamine synthetase
gloB	b0212	Hydroxyacylglutathione hydrolase
glpA	b2241	Glycerol-3-phosphate-dehydrogenase subunit anchor to FAD/NAD(P)
glpB	b2242	Glycerol-3-phosphate-dehydrogenase subunit anchor to membrane
glpC	b2243	Glycerol-3-phosphate-dehydrogenase small subunit
glpD	b3426	Glycerol 3-phosphate dehydrogenase, aerobic
glpF	b3927	Glycerol facilitator
glpK	b3926	Glycerol kinase
glpT	b2240	Glycerol-3-P Major Facilitator Superfamily (MFS)
gltA	b0720	Citrate synthase type II
gnd	b2029	6-phosphogluconate dehydrogenase
gntK	b3437	D-gluconate kinase, thermostable
gntR	b3438	GntR DNA-binding transcriptional repressor
guaC	b0104	GMP reductase
hemE	b3997	Uroporphyrinogen decarboxylase
hisB	b2022	Imidazoleglycerol-phosphate dehydratase / histidinol-phosphatase
hpt	b0125	Hypoxanthine phosphoribosyltransferase
hyaA	b0972	Hydrogenase 1 small subunit

hyaB	b0973	Hydrogenase 1 large subunit
hyuA	b2873	Phenylhydantoinase
icd	b1136	Isocitrate dehydrogenase
icdC	b4519	Isocitrate dehydrogenase (predicted protein)
idnK	b4268	Gluconate kinase
idnO	b4266	5-keto-D-gluconate 5-reductase
ilvH	b0078	Acetohydroxybutanoate synthase / acetolactate synthase, small regulatory subunit
ilvI	b0077	Acetohydroxybutanoate synthase / acetolactate synthase,large catalytic subunit
ilvM	b3769	Bifunctional acetohydroxybutanoate synthase / acetolactate synthase, large subunit
ilvN	b3670	Bifunctional acetohydroxybutanoate synthase / acetolactate synthase, small regulatory subunit
kdgK	b3526	2-keto-3-deoxygluconokinase
kdgR	b1827	KdgR DNA-binding transcriptional repressor
ldhA	b1380	D-lactate dehydrogenase
lpcA	b0222	D-sedoheptulose 7-phosphate isomerase
lpd	b0116	Lipoamide dehydrogenase; 2-oxoglutarate dehydrogenase complex and pyruvate dehydrogenase
maeA	b1479	Malic enzyme, NAD-requiring
maeB	b2463	Malic enzyme, NADP-requiring
mdh	b3236	Malate dehydrogenase
melA	b4119	α-galactosidase
mgsA	b0963	Methylglyoxal synthase
mhpF	b0351	Acetaldehyde dehydrogenase 2
nadB	b2574	L-aspartate oxidase
nanE	b3223	Predicted N-acetylmannosamine-6-phosphate epimerase
ndk	b2518	Nucleoside diphosphate kinase
nirB	b3365	Nitrite reductase small subunit
nrfA	b4070	Formate dependent nitrite reductase
pck	b3403	Phosphoenolpyruvate carboxykinase
pflA	b0902	Pyruvate formate-lyase activating enzyme
pflB	b0903	Pyruvate formate-lyase I (inactive)
pflC	b3952	Pyruvate formate-lyase activating enzyme
pflD	b3951	Formate acetyltransferase 2 predicted protein
pflF(=ybiW)	b0823	Predicted pyruvate formate lyase formate C-acetyltransferase
ppc	b3956	Phosphoenolpyruvate carboxylase
pta	b2297	Phosphate acetyltransferase / phosphate propionyltransferase
ptsG	b1101	Glucose PTS permease- PtsG subunit
purF	b2312	Amidophosphoribosyl transferase
pyrC	b1062	Dihydroorotase
pyrF	b1281	Orotidine-5'-phosphate decarboxylase
rffE	b3786	UDP-N-acetylglucosamine-2-epimerase
rpe	b3386	D-ribulose-5-phosphate 3-epimerase
rpiA	b2914	Ribose 5-phosphate isomerase, constitutive
rpiB	b4090	Ribose 5-phosphate isomerase A
sdhA	b0723	Succinate dehydrogenase flavoprotein
sdhB	b0724	Succinate dehydrogenase iron-sulfur protein

sdhC	b0721	Succinate dehydrogenase membrane protein
sdhD	b0722	Succinate dehydrogenase membrane protein
serA	b2913	α-ketoglutarate reductase / D-3-phosphoglycerate dehydrogenase
sucA	b0726	2-oxoglutarate decarboxylase, thiamine-requiring
sucB	b0727	SucB-S-succinyldihydrolipoate
sucC	b0728	Succinyl-CoA synthetase β subunit
sucD	b0729	Succinyl-CoA synthetase α subunit
talA	b2464	Transaldolase A
talB	b0008	Transaldolase B
tdcA	b3118	TdcA DNA-binding transcriptional activator
tdcB	b3117	Catabolic threonine dehydratase
tdcC	b3116	TdcC threonine/serine STP transporter
tdcD	b3115	Propionate kinase
tdcE	b3114	2-ketobutyrate formate-lyase (KFL)
tdcG	b4471	L-serine deaminase III
tdcR	b3119	TdcR DNA-binding transcriptional activator
tktA	b2935	Transketolase I
tktB	b2465	Transketolase II
yahI	b0323	Predicted carbamate kinase-like protein
ycjM	b1309	Predicted glucosyltransferase
yiaY	b3589	Predicted Fe-containing alcohol dehydrogenase
yneH	b1524	Glutaminase
zwf	b1852	Glucose-6-phosphate 1-dehydrogenase
