# Supplementary material for: Identification of enhanced hydrogen and ethanol Escherichia coli producer strains in a glycerol-based medium by screening in single-knock out mutant collections
Source: Microb Cell Fact. 2015 Jun 28;14:93. doi: 10.1186/s12934-015-0285-6 (PMC4485358; doi:10.1186/s12934-015-0285-6)
Supplement: Additional file 3: — Table S3. E. coli mutant strains with significantly higher values of specific hydrogen production (YH2/X) in blue, specific ethanol production (YE/X) in green and specific glycerol consumption (YG/X) in orange with respect to the wild type values. Intensity of colours indicates higher relative values. The 50th percentile of the values are: 14% and 13% for the YH2/X at 22 and 46 h, respectively, 47% and 33% for the YE/X at 22 and 46 h respectively, and 32% and 20% for the YG/X at 22 and 46 h respectively. The mutant strains selected according to the 50th percentile are marked in bold [file 12934_2015_285_MOESM3_ESM.pdf]

$Y_{H2/X}$			
**Mutants**	**22 h**	**Mutants**	**46 h**
frdC	0.65	***gadB***	0.21
frdB	0.59	***deoB***	0.20
gnd	0.37	***tdcE***	0.16
rpiA	0.29	***fumA***	0.16
nanE	0.26	***frmA***	0.14
frdA	0.20	***frdB***	0.14
sucA	0.19	*gcvT*	0.13
acnB	0.17	*sucC*	0.13
talB	0.14	*pflD*	0.11
tdcE	0.12	*acnB*	0.11
talA	0.12	*kdgR*	0.10
eutG	0.12	*fumB*	0.10
aldA	0.12	*frdA*	0.07
focA	0.11	*glpT*	0.07
gcvT	0.10		
sucC	0.09		
fumB	0.08		

$Y_{E/X}$			
**Mutants**	**22 h**	**Mutants**	**46 h**
frdC	0.94	***gnd***	0.63
gnd	0.67	***hyuA***	0.43
frdB	0.61	***tdcE***	0.36
rpiA	0.55	***tktB***	0.34
ldhA	0.52	*gntR*	0.34
idnK	0.52	*eutG*	0.34
hyuA	0.48	*yiaY*	0.33
tdcE	0.46	*idnO*	0.33
gntK	0.40	*ldhA*	0.33
frdA	0.39	*idnK*	0.32
kdgK	0.35	*kdgR*	0.30
tktA	0.31	*glpF*	0.29
ilvI	0.29	*dacA*	0.28
glgC	0.21		

$Y_{Glic/X}$			
**Mutants**	**22 h**	**Mutants**	**46 h**
nanE	1.04	***gnd***	0.37
frdC	0.81	***frmA***	0.36
rffE	0.76	***rffE***	0.33
frdB	0.59	***cpsG***	0.32
rpiA	0.46	***fabH***	0.29
gnd	0.35	***galK***	0.28
cpsG	0.34	***glgC***	0.28
tdcE	0.33	***tdcE***	0.21
galK	0.31	***deoB***	0.20
glgC	0.31	***nanE***	0.20
idnK	0.28	*aldA*	0.17
ldhA	0.28	*frdC*	0.17
gntK	0.24	*sucC*	0.17
tdcB	0.21	*eutG*	0.14
kdgK	0.15	*PFLD*	0.14
fdoH	0.14	*tktB*	0.13
		yiaY	0.12
		aceF	0.12
		fumA	0.10
