# Supplementary material for: Identification of enhanced hydrogen and ethanol Escherichia coli producer strains in a glycerol-based medium by screening in single-knock out mutant collections
Source: Microb Cell Fact. 2015 Jun 28;14:93. doi: 10.1186/s12934-015-0285-6 (PMC4485358; doi:10.1186/s12934-015-0285-6)
Supplement: Additional file 5: — Table S4. Relative hydrogen and ethanol production in E. coli mutant strains using different carbon sources and pH. [file 12934_2015_285_MOESM5_ESM.docx]

| **Mutant strains** | **Relative hydrogen production^c^** | **Relative ethanol production^c^** | **Carbon source** | **Reference** |
| --- | --- | --- | --- | --- |
| MG1655 ΔfrdA::FRTΔptA::tetR | 1.06 | 1.11 | Glycerol, tryptone | Yazdani and Gonzalez, 2008^b^ |
| BW25113Δ*frdA*Ωkan^R^ | 1.15 | 1.17 | Glycerol, peptone | This work^b^ |
| BW25113Δ*frdC*Ωkan^R^ | 1.8 | - | Glycerol | Tran et al., 2014^a^ |
| BW25113Δ*frdC*Ωkan^R^ | 1.4 | 1.3 | Glycerol, peptone | This work^b^ |
| BW25113Δ*ldhA*Ωkan^R^ | 1.1 | - | Glycerol | Tran et al., 2014^a^ |
| BW25113Δ*ldhA*Ωkan^R^ | 1.15 | 1.2 | Glycerol, peptone | This work^b^ |
| K1060 Δ*ldhA*Ωkan^R^ | - | 1.5 | Glycerol, yeast extract, amino acids | Nikel et al., 2010^a^ |
| W3110 Δ*ldhA*::Cm^R^, *frdBC*::Km^R^ | 1.4 | 1.5 | Glucose, yeast extract, tryptone peptone | Yoshida et al., 2006^b^ |
| BW25113Δ*gnd*Ωkan^R^ | 1.3 | 1.33 | Glycerol, peptone | This work^b^ |
| BW25113Δ*tdcE*Ωkan^R^ | 1.1 | 1.15 | Glycerol, peptone | This work^b^ |

^a^Experiments were conducted at pH 7.5 and samples were taken at 24 h.

^b^Experiments were conducted at pH 6.25-6.3 and samples were withdrawn at 22 h.

^a,b^Both experiments were carried anaerobically with normal pressure.

^c^Parameters of hydrogen and ethanol production values expressed in different ways (µmol/mg protein; mmol/ g of CDW; mmol/ g CDW/h) are relativized with the wild type ones.
