# Supplementary material for: Identification of enhanced hydrogen and ethanol Escherichia coli producer strains in a glycerol-based medium by screening in single-knock out mutant collections
Source: Microb Cell Fact. 2015 Jun 28;14:93. doi: 10.1186/s12934-015-0285-6 (PMC4485358; doi:10.1186/s12934-015-0285-6)
Supplement: Additional file 8: — Table S2. The primers from 1 to 6 were used to obtain multiple mutants; ldhAgnd:kan (M2), ldhAgndfrdBC::kan (M4) and ldhAgndfrdBCtdcE::kan (M5). Capital letters indicate the homologous sequences of the target genes and in lower case letters indicate the priming sequences of the pKD13 vector. In the names of the primers, H2P1 indicates the forward primers and H1P4 the reverse primers. Primers from 7 to 15 were used for PCR verification of the deletion of the targeted genes. [file 12934_2015_285_MOESM8_ESM.pdf]

Primers	Sequences
H1P4-gnd	CCATTCAGCGCGGTGATCACACCTGACAGGAGTATGTAATGattccggggatccgtcgacc
H2P1-gnd	TATTGTTGGTTAAATCAGATTAATCCAGCCATTCGGTATGtgtaggctggagctgcttcg
H1P4-frdBC	GGATGCAGCCGATAAGGCGGAAGCAGCCAATAAGAAGGAGAAGGCGAATGattccgg ggatccgtcgacc
H2P1-frdBC	TTGGATTTGGATTAATCATCTCAGGCTCCTTACCAGTACAGGGCAACAAAtgtaggctgg agctgcttcg
H1P4-tdcE	ATAATTTAGTTGAAGTATTGTAGAGAGATTATTTTTCATGattccggggatccgtcgacc
H2P1-tdcE	TCATACATCCTCCGGCGTCAGAGCGCCTGGGTAAAGGTTGtgtaggctggagctgcttcg
ldhA-F	TGGCGATTGCTCCGTCTGCG
ldhA-R	GCGGTCGCCAGCGTTAACTG
gnd-F	TTGTGCGTGTAAATGGCTTCG
gnd-R	GGATCATAGTCGGTTGGAGTG
frdA-F	ACGAAGTCTACTCGCAACGC
frdD-R	CCGATACTGGAGTTGGCATAC
tdcE-F	TCTGGTCATGGAACATTTGGC
tdcE-R	GGCTCGTCGCGGTTAATAAGC
kt	CGGCCACAGTCGATGAATCC
